# The Impact of Educational Attainment on Observed Race/Ethnic Disparities in Inflammatory Risk in the 2001–2008 National Health and Nutrition Examination Survey

**DOI:** 10.3390/ijerph13010042

**Published:** 2015-12-22

**Authors:** Gniesha Y. Dinwiddie, Ruth E. Zambrana, Lauren A. Doamekpor, Lenny Lopez

**Affiliations:** 1 African American Studies Department, University of Maryland, College Park, MD 20742, USA; 2 Maryland Population Research Center, University of Maryland, College Park, MD 20742, USA; 3 Women’s Studies Department, University of Maryland, College Park, MD 20742, USA; rzambran@umd.edu; 4 Consortium on Race, Gender and Ethnicity, University of Maryland, College Park, MD 20742, USA; 5 National Center for Health Research, Washington, DC 20036, USA; doamekpor3@gmail.com; 6 Department of Medicine, University of California, San Francisco, CA 94121, USA; Lenny.Lopez@ucsf.edu

**Keywords:** C-reactive protein, education, inflammation burden, race/ethnicity

## Abstract

Inflammation has shown to be an independent predictor of cardiovascular disease (CVD) and growing evidence suggests Non-Hispanic Blacks (NHBs) and certain Hispanic subgroups have higher inflammation burden compared to Non-Hispanic Whites (NHWs). Socioeconomic status (SES) is a hypothesized pathway that may account for the higher inflammation burden for race/ethnic groups yet little is known about the biological processes by which SES “gets under the skin” to affect health and whether income and education have similar or distinct influences on elevated inflammation levels. The current study examines SES (income and education) associations with multiple levels of C-Reactive Protein (CRP), an important biomarker of inflammation, in a sample of 13,362 NHWs, 7696 NHBs and 4545 Mexican Americans (MAs) in the United States from the 2001 to 2008 National Health and Nutrition Examination Survey. After adjusting for age, sex, and statin use, NHBs and MAs had higher intermediate and high CRP levels compared to NHWs. Income lessened the magnitude of the association for both race/ethnic groups. The greater intermediate and high CRP burden for NHBs and MAs was strongly explained by educational attainment. MAs were more vulnerable to high CRP levels for the lowest (*i.e.*, less than nine years) and post high school (*i.e.*, associates degree) educational levels. After additional adjustment for smoking, heavy drinking, high waist circumference, high blood pressure, diabetes and statin use, the strength of the association between race/ethnicity and inflammation was reduced for NHBs with elevated intermediate (RR = 1.31; *p* ≤ 0.001) and high CRP levels (RR = 1.14; *p* ≤ 0.001) compared to NHWs but the effect attenuated for MAs for both intermediate (RR = 0.74; *p* ≤ 0.001) and high CRP levels (RR = 0.38; *p* ≤ 0.001). These findings suggest educational attainment is a powerful predictor of elevated CRP levels in race/ethnic populations and challenges studies to move beyond examining income as a better predictor in the SES-inflammation pathway.

## 1. Introduction

Cardiovascular disease (CVD) is the leading cause of death in the United States. Recent health guidelines reinforce the importance of cardiovascular health promotion and primary prevention since it is a major cause of disability, contributes to lower life expectancy, and a burden on the health care system [1,2,3]. National estimates indicate CVD prevalence rates are highest for Non-Hispanic blacks (NHBs) (45% for both sexes), compared to Non-Hispanic whites (NHWs) (38% for men and 33% for women) and Hispanics (26% for men and 32% for women). However, there is variation by Hispanic subgroup where Mexican Americans have the highest CVD risk [4,5,6].

Increasing evidence demonstrates that biological risk indicators, such as C-reactive protein (CRP), can predict the presence, susceptibility, and physiologically related assessments for CVD [7,8,9]. CRP, an acute phase protein considered a marker of systemic inflammation, has been shown to be an independent predictor of stroke, myocardial infarction, atherosclerosis, peripheral vascular disease and sudden cardiac death [10,11,12,13,14]. Known differences in CRP levels have been reported by race/ethnicity [15] with higher CRP levels for NHBs and Hispanics [16,17] compared to NHWs, but the etiology of the risk factors driving these differences is unclear [18].

Multiple social, behavioral and biological pathways are hypothesized as important predictors of increased CRP levels for race/ethnic groups. However, socioeconomic status (SES) as a predictor of CRP levels has shown inconsistent positive associations. SES, a dynamic social pathway, includes income, education, and occupation that possibly mediate health risks. Education is hypothesized to have a unique impact on health because it’s unaffected by health impairments that may emerge in adulthood (which impact income) and it allows one to move up the wealth gradient by obtaining access to occupations that pay higher wages. Since non-Whites tend to have lower educational attainment and income than Whites, SES as measured by educational attainment, can reasonably be considered a potential mediating pathway in the association between race/ethnicity and elevated CRP levels [19]. To date, the association of SES and CRP has been assessed in NHBs in comparison to NHWs and have produced mixed, empirical results [20,21,22]. For example, Koster *et al.* [20] report similar SES-inflammation associations in NHB and NHW adults whereas other studies find no SES related Black/White differences [21,22]. Educational attainment is an important SES component that has not been fully explored for race/ethnic groups. Of the few studies that have investigated racial/ethnic group outcomes, an inverse relationship between educational attainment and CRP levels was shown for NHWs but not NHBs [15], while higher levels of education were associated with increased CRP levels for Hispanics [23]. Investigations of educational pathways with elevated CRP for multiple race/ethnic groups in one investigation have found NHWs and NHBs with higher educational attainment have lower inflammation levels but not Hispanics [24]. Another study found that educational attainment was not associated with elevated CRP levels for NHBs compared to NHWs and Hispanics [25].

Further examination of the educational pathway in the race/ethnicity–CRP association may inform our understanding of the biological pathways through which education impacts health in different demographic groups [15]. Findings can add to the existing literature on racial/ethnic disparities as these groups have a greater burden of diseases in which inflammatory processes play a role (*i.e.*, obesity, hypertension, diabetes) and may engage in adverse health behaviors that increase inflammation levels (*i.e.*, smoking and heavy drinking) [26,27]. However, smoking and heavy drinking have not consistently explained elevated CRP levels for NHBs or Hispanics [28,29].

This study aims to test whether hypothesized educational pathways underlie disparities in CRP levels for NHWs, NHBs and Mexican Americans (MAs) in the National Health and Nutrition Examination Survey (NHANES). Increasing evidence shows that SES indicators such as income and education have different associations with health [30,31] such that the bulk of the relationship for income is concentrated in low income categories experiencing the highest burden of adverse health whereas there might be an educational gradient where greater differences in health appear at higher educational levels in comparison to lower levels [32]. We expect similar patterns of associations of lower educational attainment and elevated CRP levels for race/ethnic groups understudy. In addition, we focus our analysis on MAs who are the largest U.S. Hispanic subgroup represented in NHANES. In this paper, we examine SES as a primary exposure rather than adjusting for SES and build on prior studies that have found conflicting associations between SES and elevated CRP in race/ethnic groups. Given their disproportionate representation in lower SES categories, we hypothesize that NHBs and MAs will have higher CRP levels compared to NHWs by level of inflammation (*i.e.*, intermediate or high risk). Furthermore, we expect that NHBs and MAs will have elevated CRP levels as educational levels surpass high school diploma.

## 2. Methods

Pooled data were obtained from the 2001 to 2008 NHANES, a cross-sectional survey designed to assess health and nutritional status of adults in the United States. The survey includes information on demographic, socioeconomic, dietary, medical, dental, physiological measurements, and laboratory tests administered by trained medical personnel [33]. This study examines data from respondents who underwent medical examinations and completed a battery of laboratory tests including blood specimens for testing CRP. The study uses a complex sampling design consisting of sampling at the county, household and individual levels. The NHANES oversamples race/ethnic groups for a representative sample. The NHANES protocol was approved by a governmental Institutional Review Board. All respondents were compensated with a financial incentive for their participation.

The sample includes 13,262 NHWs, 7,696 NHBs, and 4,545 MAs born in one of the 50 states or Washington D.C. over 20 years of age. Analyses were weighted with the Mobile Examination Center sampling weight to account for the complex design of pooled data. We adjusted the weight by dividing by the number of survey years to obtain the average U.S. civilian non-institutionalized population to generalize our findings to the U.S. population.

CRP level is the dependent variable, measured by a high sensitivity assay using latex-enhanced nephelometry, with a lower limit of detection of 0.1 mg/L. Blood specimens were processed, stored, and shipped to the Johns Hopkins University Lipoprotein Analytical Laboratory for analysis. Details of sample collection, measurement procedures, quality control, and quality assurance have been described elsewhere [33]. We chose to focus on multiple CRP levels since most studies have relied upon a continuous or dichotomous measure to represent variations in CVD risk that may be related to different sources of risk with either chronic or acute implications [34]. These levels are used in clinical settings to determine inflammation risk, and intervene at critical stages to prevent the risk and progression of CVD and its sequele, therefore CRP was coded into 3 clinically relevant categories: low ≤1 mg/L, intermediate = 1–3 mg/L, and high ≥3 mg/L [35,36]. Participants with CRP levels ≥10 mg/L (*n* = 10) were excluded from the analysis since this level typically signals acute illness.

Race, demographic, SES, health behavior and health condition variables were obtained directly from the NHANES and not recoded. Self-reported race/ethnicity was ascertained from respondents (*i.e.*, NHWs, NHBs, MAs). Since race/ethnicity is a multidimensional category, we are not measuring race/ethnicity as an independent variable, but rather assessing how racial stratification exposes these groups to additional risk factors that induce health risks [37]. Demographic variables included age, (20–34, 35–44, 45–54, 55–64, or 65+ years) and sex (men, women). Income was categorized as 0–$24,999, $25,000–$44,999, $45,000–$74,000, or $75,000 and above. Educational attainment includes <9 years of schooling, 9 to 11 years, a high school diploma/GED, some college/AA degree, or post-secondary degree or higher. Health behaviors include current smokers based on self-reporting (yes, no), and heavy drinkers consuming five or more alcoholic beverages per day (yes, no). Health conditions were measured by a question that asked respondents “Ever been told by a doctor or health professional that {you have/{he/she/SP} has} Diabetes or sugar Diabetes (1 = yes, 0 = no) or high blood pressure or high blood sugar (1 = yes, 0 = no). To assess obesity, waist circumference was measured using a fiberglass tape crossing over the umbilicus. Waist circumference has been shown to be a more robust measure of CVD risk for NHBs and MAs [38,39,40]. Cut-off points are 88 cm for women and 102 cm for men then transformed into a dichotomous variable (yes, no). Statin use was obtained by a questionnaire that asked respondents “In the past month, have you used or taken medication for which a prescription is needed?” If the respondent answered “yes” then the respondent provided the name of the medication that was verified by the interviewer. NHANES provides drug codes for all medications and we selected drugs used in NHANES for statins. Selected drug names were verified by a cardiologist (*i.e.*, atorvastatin, cerivastatin, fluvastatin, lovastatin, pravastatin, and simvastatin) then transformed into a dichotomous variable to indicate statin use (1 = yes, 0 = no). Although more recent data from NHANES is available, the prescription file for 2010 did not include statins for public use. Therefore due to data constraints, we use pooled data from 2001 to 2008. Interaction terms tested associations between race/ethnicity and SES predictors.

SAS version 9.2 is used to perform the weighted analyses (SAS Institute, Inc., Cary, North Carolina). Descriptive statistics are presented as weighted proportions. Chi-square tests are used to test levels of significance in descriptive analyses. In our sensitivity analyses, we treated the response variable as dichotomous, placing those with CRP levels ≥3 mg/L into the “high” category. Our results showed MA’s were more likely to have “high” CRP levels, earning an SC/AA degree was associated with being in a “High” CRP category, and low income was positively associated with high CRP. In the current analysis, we employed weighted multinomial logistic regression models assessing the relative risk of being in an intermediate or high CRP level relative to low risk. These relative risks are also referred to as odds ratios and calculate the log odds that a member of group, falls into a category, as opposed to the reference group. Model 1 assesses the direct association between race/ethnicity on CRP levels with NHWs as the reference category. This step tests whether there are race/ethnic differences in CRP levels and the magnitude of these differences. Model 2 incorporates income to examine if this pathway explains or attenuates the effect for NHBs and MAs. Model 3 introduces education. Income and education were tested in different steps in order to parcel out the different impact of these two important measures of SES on CRP levels. Educational attainment is entered in Model 3 since we wanted to control for known factors that could confound the model for education since it is our main SES mechanism understudy. The full model (model 4) includes controls for health behaviors, health status, statin use and significant interactions to assess the best model that may explain race/ethnic disparities in elevated CRP levels. To investigate whether an educational gradient exists, we estimated predicted probabilities over educational attainment by race/ethnicity for low, intermediate and high CRP levels. This step allows for the estimation of predicted probabilities for each educational level while holding income, sex, age, health behaviors, health status and statin use constant.

## 3. Results

Table 1 shows weighted descriptive statistics of the sample. Three quarters of NHWs and over 60% of NHBs were over 35 years old, whereas half of MAs were in the youngest age category (20–34 years). The sample was advantaged by females for all three race/ethnic groups. In important SES categories, income disparities were evident between race/ethnic groups. The majority of NHWs were represented in the highest category compared to the majority of NHBs and MAs who made less than $25,000 per year.

Surprisingly, educational disparities were less pervasive. Over one third of NHWs, NHBs and MAs had some college training or an Associate’s Degree. However, more NHWs (29%) compared to NHBs (16%) and MAs (12%) had a bachelor’s degree, which is an important SES indicator associated with higher income, more stable job opportunities and sustainable lifestyles. Results also show that over half of NHBs were smokers compared to NHWs (41%) and MAs (36%). Low percentages of heavy drinkers were observed for all three groups. Mexican Americans represent the group with the highest percent of normal waist circumference (49%) compared to 34% of NHWs and 41% of NHBs. For health conditions, nearly 20 percent of NHBs had hypertension, and 10% had diabetes. The majority of NHWs, NHBs and MAs were not using statins but more NHWs (11%) were using statins compared to 6% of NHBs and 4% of MAs. Although the percent distribution on CRP shows the majority of race/ethnic groups fall in the low level category, NHBs have higher representation in both the intermediate and high CRP levels.

**Table 1:** Weighted sample descriptive statistics by race/ethnicity, national health and nutrition examination survey, United States, 2001–2008.

Variable	Non-Hispanic White *n* = 13,362	Non-Hispanic Black *n* = 7696	Mexican American *n* = 4545	*p*-Value
Age				
20–34	26.7%	36.8%	49.6%	*p* ≤ 0.001
35–44	19.2	20.3	17.5	
45–54	21.7	19.8	14.7	
55–64	13.6	11.7	9.3	
65+	18.8	11.3	8.9	
Sex				
Female	50.9%	54.0%	51.3%	*p* ≤ 0.001
Male	49.1	46.0	48.7	
Income				
Less than 25,000	18.7%	37.9%	31.1%	*p* ≤ 0.001
25,000–44,999	21.3	26.0	28.1	
45,000–74,000	25.1	19.3	22.2	
75,000+	34.9	16.8	18.5	
Education				
Less than 9 years	3.6%	5.3%	8.5%	*p* ≤ 0.001
9–11 years	9.4	22.7	19.5	
High school diploma/GED	26.8	24.2	26.1	
Some college/AA degree	31.6	32.3	33.5	
Post Secondary or higher	28.7	15.5	12.4	
Health Behaviors				
Non Smoker	59.4%	45.9%	63.9%	*p* ≤ 0.001
Smoker	40.6	54.1	36.1	
Not Heavy Drinker	83.9	83.7	85.2	
Heavy drinker	16.1	16.3	14.8	
Health Status				
Normal Waist Circumference	34.1%	40.6%	49.6%	*p* ≤ 0.001
High Waist Circumference	65.9	59.4	50.4	
Normal Blood Pressure	84.2	81.0	90.8	
High Blood Pressure	15.8	19.0	9.2	
Diabetic	6.8	9.6	5.7	
Statin User	11.2	6.1	3.5	
C-Reactive Protein				
Low	92.2%	88.0%	90.0%	*p* ≤ 0.001
Intermediate	6.7	10.6	7.9	
High	1.2	1.4	1.3	

Note: All statistics represent column percent. Statistics between race/ethnicities are significant at the *p* < 0.001 level.

Weighted descriptive statistics of the predictor variables with CRP levels are shown in Table 2. More females than males were in intermediate and high CRP level categories. Non-Hispanic Blacks had higher representation in both elevated CRP categories compared to NHWs and MAs. Ten percent of respondents aged 55–64 were represented in the intermediate CRP category. For important indicators of socioeconomic status, a higher percentage of respondents in the lowest income category were represented in both intermediate and high CRP level categories. However, for respondents with 9–11 years of schooling, 13.2% had intermediate CRP and 2.2% had high CRP levels. Although the majority of the sample that practiced adverse health behaviors such as cigarette smoking and heavy drinking had low CRP levels, almost 10% of smokers were represented in the intermediate CRP category and 2% had high CRP levels. In contrast to smokers, a higher percent of respondents that were not heavy drinkers (9%) had intermediate CRP levels. Lastly, the distribution of health conditions show 10% of respondents with HWC and HBP had intermediate CRP levels, whereas 15% of the population with diabetes were in this category. Only 2% of diabetics had high CRP levels.

**Table 2:** Weighted descriptive statistics by C-reactive protein level.

Variable	Low CRP	Intermediate CRP	High CRP	*p*-Value
Sex				
Female	88.8%	9.8%	1.4%	*p* ≤ 0.001
Male	94.4	4.6	1.0	
RACE				
NHW	92.2%	6.7%	1.2%	*p* ≤ 0.001
NHB	88.0	10.6	1.4	
MA	90.9	7.9	1.3	
AGE				
20–34	92.0%	6.8%	1.2%	*p* ≤ 0.001
35–44	91.6	7.3	1.1	
45–54	88.9	9.8	1.2	
55–64	88.4	10.3	1.4	
65+	88.8	9.1	2.2	
Income				
less than 25,000	88.8%	9.7%	1.6%	*p* ≤ 0.001
25,000–44,999	90.9	7.6	1.4	
45,000–74,000	91.4	7.4	1.2	
75,000+	93.8	5.4	0.8	
Education				
less than 9 years	86.8%	10.8%	2.4%	*p* ≤ 0.001
9–11 years	84.6	13.2	2.2	
HS	88.8	10.0	1.2	
Some College	90.5	8.1	1.4	
Post-Secondary	90.1	8.5	1.4	
Health Behaviors				
Non smokers	89.7%	8.9%	1.4%	*p* ≤ 0.001
Smokers	88.6	9.7	1.7	
Not Heavy Drinkers	89.9	8.8	1.3	
Heavy Drinkers	91.3	7.3	1.4	
Health Condition				
Normal waist	97.0%	2.2%	0.7%	*p* ≤ 0.001
HWC	88.9	9.7	1.4	
Normal Bldpres	92.0	6.8	1.1	
High Bldpres	88.3	10.4	1.3	
Non Diabetic	92.2	6.7	1.1	
Diabetic	82.4	15.2	2.4	

Abbreviations: NHW, Non-Hispanic White; NHB, Non-Hispanic Black; MA, Mexican American; HWC, High waist circumference; Bldpres, Blood Pressure; HS/GED, High School/GED; SC/AA, Some College/Associate’s Degree. All row statistics are significant at the *p* < 0.001 level.

Table 3 shows results from multinomial logistic regression assessing the association between race/ethnicity and intermediate and high CRP levels relative to low CRP adjusting for age, sex, health behaviors, health status and statin use. The relative risk for being in an intermediate CRP level is 1.66 times higher for NHBs compared to NHWs (Model 1). Similar to NHBs, MAs had higher intermediate CRP levels (RR = 1.20; *p* < 0.001) compared to NHWs. Also, NHBs (RR = 1.38, *p* < 0.001) and MAs (RR = 1.10, *p* < 0.001) had greater high CRP levels compared to NHWs (Model 1). When income was included in the model (Model 2), the relative risk of being in an intermediate CRP level slightly decreased for NHBs (RR = 1.52; *p* < 0.001) but marginally increased for MAs (RR = 1.12; *p* < 0.001). Respondents with incomes ≤$25,000 had the highest intermediate inflammation levels in comparison to respondents making over $75,000 per year. For high CRP levels (Model 2), relative risk ratios decreased from the previous model for NHBs (RR = 1.20; *p* < 0.001) and MAs (RR = 1.05; *p* < 0.001). Similar to intermediate levels, respondents with incomes ≤$25,000 had the greatest risk for high CRP levels. When educational attainment was entered into the additive model (Model 3), NHBs (RR = 1.69; *p* < 0.001) and MAs (RR = 1.47; *p* < 0.001) showed higher intermediate CRP levels compared to NHWs and the magnitude of those associations strengthened from the previous model (Model 2). In addition, the predicted odds of intermediate CRP levels were 1.58 times higher for respondents with 9–11 years of schooling. In the full model (Model 4) that adjusts for health behaviors, health status, age, and statin use, findings show NHBs had higher intermediate CRP levels (RR = 1.31; *p* < 0.001*)* compared to NHWs, but the strength of that association decreases from the previous model. However, the effect for MAs disappeared (RR = 0.74; *p* < 0.001). For high CRP, Model 4 shows the predicted odds of high CRP was 1.14 times higher for NHBs (RR = 1.14; *p* < 0.001) but lower for MAs (RR = 0.38: *p* < 0.001). Interactions reveal race/ethnic effects are dependent on education for intermediate CRP levels in NHBs, and lower income for intermediate CRP levels in MAs. Interactions for high CRP level show the race/ethnicity effect for NHBs is contingent on educational attainment, particularly the 9–11 years of schooling category.

**Figure 1:**
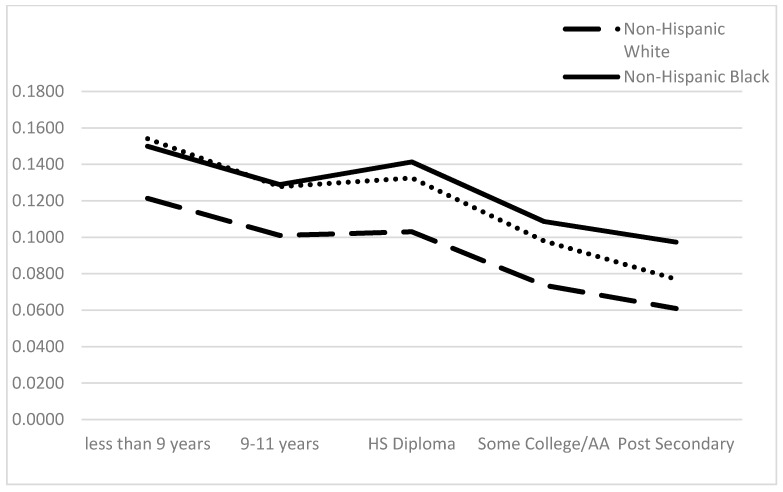
Predicted Probability of Intermediate C-Reactive Protein by Education and Race/Ethnicity. Note: Differences Across Groups Statistically significant at the *p* < 0.001 level. Model controls for age, diabetes, heavy drinker, HBP, HWC, income, sex, smoker, and statin use.

**Figure 2:**
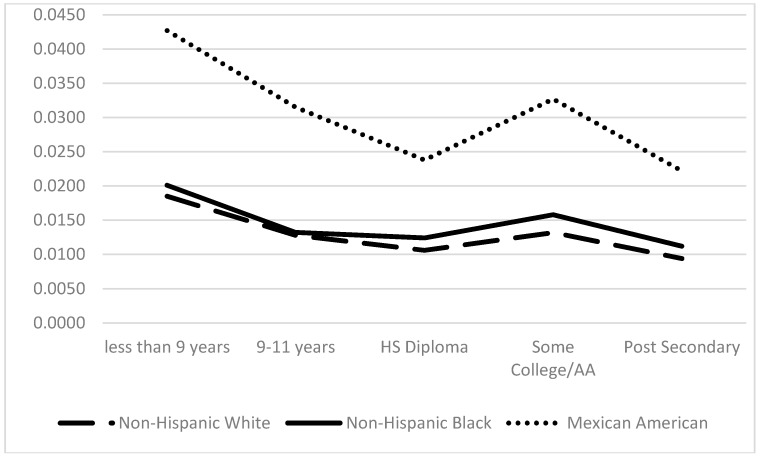
The predicted probability of high C-reactive protein by education and race/ethnicity. Note: Differences across groups statistically significant at the *p* < 0.001 level. Model controls for age, sex, income, education, smokers, heavy drinkers, HBP, HWC, diabetes, statin use and interactions.

Secondary analyses examining whether an educational gradient exists for race/ethnic groups on CRP levels are represented in Figure 1 and Figure 2. For intermediate CRP level, NHWs have more favorable profiles at every educational category compared to NHBs and MAs (Figure 1). Both NHBs and MAs with ≤9 years of schooling display a 16% higher probability of being in an intermediate CRP level. However, 9–11 years of schooling proves to be an important marker of intermediate CRP level since the probability of risk increases for NHBs for this educational category whereas for MAs, intermediate CRP levels decline with more educational attainment. Overall, MAs have the highest probability of high CRP at all levels of educational attainment (Figure 2). Instead of a clear educational gradient, trajectories show the probability of high CRP levels decrease for MAs that have ≤ a high school diploma, then increases for MAs with some college or an Associate’s degree. NHWs and NHBs show similar high CRP patterns across educational attainment.

**Table 3:** Multinomial regression models for intermediate and high levels of C-reactive protein by race/ethnicity, national health and nutrition examination survey, United States, 2001–2008.

	Model 1		Model 2		Model 3		Model 4	
	CRP Level							
	1–3 mg/dL (95% CI)	>3 mg/dL (95% CI)	1–3 mg/dL (95% CI)	>3 mg/dL (95% CI)	1–3 mg/dL (95% CI)	>3 mg/dL (95%CI)	1–3 mg/dL (95% CI)	>3 mg/dL (95% CI)
**Race/ethnicity**								
NHW(ref.)								
NHB	1.66 (1.65–1.66)	1.31 (1.30–1.31)	1.52 (1.52–1.52)	1.20 (1.19–1.21)	1.69 (1.69–1.70)	1.36 (1.35–1.36)	1.31 (1.30–1.32)	1.14 (1.13–1.15)
MA	1.20 (1.19–1.20)	1.10 (1.09–1.11)	1.12 (1.11–1.12)	1.05 (1.05–1.06)	1.47 (1.46–1.47)	1.51 (1.50–1.52)	0.74 (0.73–0.75)	0.38 (0.37–0.39)
**Income**								
Less than 25,000			1.76 (1.76–1.77)	1.99 (1.98–2.00)	1.49 (1.48–1.49)	2.06 (2.05–2.07)	1.24 (1.23–1.24)	3.51 (3.47–3.54)
25,000–44,999			1.41 (1.40–1.41)	1.80 (1.78–1.80)	1.16 (1.15–1.16)	1.91 (1.90–1.92)	1.06 (1.05–1.06)	3.54 (3.51–3.57)
45,000–74,000			1.39 (1.39–1.39)	1.48 (1.47–1.49)	1.22 (1.22–1.22)	1.58 (1.57–1.58)	1.11 (1.11–1.12)	1.75 (1.73–1.76)
75,000+ (ref.)								
**Education**								
Less than 9 years					1.32 (1.36–1.37)	1.48 (1.47–1.49)	1.91 (1.90–1.92)	1.13 (1.12–1.15)
9–11 years					1.58 (1.57–1.59)	1.30 (1.29–1.30)	1.49 (1.49–1.50)	0.70 (0.69–0.71)
HS/GED					1.54 (1.53–1.54)	0.84 (0.83–0.84)	1.55 (1.54–1.55)	0.77 (0.77–0.78)
SC/AA degree					1.25 (1.25–1.26)	0.99 (0.98–0.99)	1.06 (1.05–1.06)	1.05 (1.03–1.05)
Post-Secondary or Higher (ref.)								
**Controls**								
**Sex** (1 = male)							0.45 (0.45–0.46)	0.58 (0.57–0.58)
**Age**								
20–34							0.91 (0.91–0.92)	0.39 (0.39–0.40)
35–44							0.94 (0.97–0.98)	0.33 (0.32–0.33)
45–54							1.52 (1.52–1.53)	0.54 (0.53–0.55)
55–64							1.04 (1.03–1.04)	0.61 (0.60–0.61)
65+ (ref.)								
**Health behaviors**								
Smokers (Yes)							1.20 (1.19–1.20)	1.66 (1.65–1.67)
Heavy drinkers (Yes)							0.89 (0.88–0.89)	1.08 (1.07–1.09)
**Health Status**								
HWC							3.22 (3.20–3.22)	1.31 (1.30–1.32)
HBP							1.04 (1.03–1.04)	0.72 (0.72–0.74)
Diabetes							1.78 (1.77–1.79)	0.82 (0.81–0.82)
Statin (Yes)							1.01 (1.00–1.01)	1.01 (0.99–1.01)

Abbreviations: NHW, Non-Hispanic White; NHB, Non-Hispanic Black; MA, Mexican American; HWC, High waist circumference; HS/GED, High School/GED; SC/AA, Some College/Associates Degree; CI, Confidence Interval; Ref, Reference Group. Model 1: Race/Ethnicity; Model 2: Race/Ethnicity, Income; Model 3: Race/Ethnicity, Income, Education; Model 4: Race/Ethnicity, Income, Education, Health Behaviors, Health Status, Statin Use, Interactions, Age, Sex.

## 4. Discussion

Using a nationally representative cohort sample in the United States, significant racial/ethnic disparities in CRP levels were found to be strongly associated with educational attainment. In the current study, education consistently strengthened the relationship between race/ethnicity and CRP level, showing more explanatory power compared to income. In support of our first study expectation and consistent with a growing body of research, our data extend and confirm prior findings of elevated CRP levels for NHBs and Hispanics that were explained by lower educational attainment [24,25] but extend this area to include MAs, an important Hispanic subgroup. In contrast to previous studies that suggest similar [20] or no race/ethnic differences [15,21,22] in education-inflammation associations, our results confirm the strength of directly measuring education level, over income, among minorities to enhance its analytic predictive power. Future studies should account for the importance of the educational component in the SES pathway to understand race/ethnic disparities in inflammation burden.

In this study, educational attainment as a SES indicator, represents an important dynamic social mechanism that shows a unique association with CRP levels. Therefore, education may be a more stable and reliable social pathway relative to income since the majority of Americans have had some exposure to schooling; are unaffected by health impairments that may emerge in adulthood; and have the potential to increase income to purchase health-enhancing goods such as health care services, nutritious food, residence in cleaner environments, reduce health risk behaviors, and decrease stress [41,42]. Our findings confirm these differential effects by showing that low educational attainment is associated with intermediate (*i.e.*, 9–11 years of schooling) and high CRP levels (*i.e.*, less than 9 years of schooling) in the SES-race/ethnicity pathway for both NHBs and MAs.

Our results show that income has an attenuating effect on intermediate and high CRP levels for both NHBs and MAs. Many correlational studies of income and health that are stratified by race/ethnicity normally report stronger associations with income for Whites [43]. Explanations for this effect for our race/ethnic groups might suggest that income is not strongly associated with all indicators of health as previously assumed.

Our results show health behaviors such as cigarette smoking were positively associated with both intermediate and high CRP levels, whereas high CRP levels were greater for heavy drinkers. Positive associations were found between HWC and both intermediate and high CRP levels. In addition, HBP and diabetes were associated with intermediate CRP levels. However, these health behavior and health condition pathways did not explain race/ethnic disparities in CRP levels as the strength of the association lessened for NHBs and disappeared for MAs when entered into the full models. Similar to our findings, Paalani *et al.* [44] found high CRP levels were attenuated for NHBs when controlling for SES, exercise, diet, smoking, alcohol consumption, waist circumference, diabetes, stroke and sleep apnea. This study shows that health behaviors and health conditions lessen the effect for NHBs at both intermediate and high CRP levels, making a significant contribution to the literature. Lastly, our findings challenge studies that report high waist circumference as a significant predictor of elevated CRP levels for Mexicans [45] as the present study showed health conditions may not fully explain elevated inflammation levels for this important population considering the overwhelming evidence that educational attainment does.

There is growing scientific literature that demonstrates that Hispanics and Non-Hispanic Blacks are more likely to have elevated inflammatory biomarkers compared to non-Hispanic whites where differences in CRP levels by racial/ethnic group are not entirely explained by traditional CVD risk factors, suggesting that environmental or genetic influences may also be operative [17]. In our predicted probability models, our hypothesis that NHBs and MAs will have greater elevated CRP levels compared to NHWs as educational attainment surpasses a high school diploma was partially supported for NHBs with intermediate and MAs with high CRP levels. Education’s unique impact on health is hypothesized to have a differential effect compared to income, where there may be greater differences between college graduates and high school graduates relative to those with a high school diploma or below [46]. Our study showed NHBs had greater intermediate CRP levels beginning with 9–11 years of schooling and beyond, which contradicts previous findings that suggest an inverse relationship exists between educational attainment and inflammation [32]. A likely explanation for this finding is that the returns of educational attainment work differently in NHB populations such that education does not operate as a buffer to protect against disease risk. Moreover, a novel finding that MAs are more vulnerable to high CRP at all educational levels is a noteworthy contribution. Prior studies have found inconsistent SES-health gradients in Hispanics, sometimes showing a flattened or even a reversed pattern that may vary by national origin, nativity, or acculturation [30,47,48]. The existing studies elucidating the relationship between ethnicity and inflammation have called for researchers to disaggregate the Hispanic category and to investigate the predictive value of multiple CRP levels that may vary by national origin, nativity, and acculturation [24]. We contribute to this body of evidence by providing data that show variations in the educational predictors associated with intermediate and high CRP levels for MAs who represent 65% of Hispanics, and are concentrated in environments which may increase exposure to higher rates of depression, discrimination, racism, and other sources of psychological stress; each of these factors has been independently shown to affect CRP levels and subsequent cardiovascular disease risk [49]*.* Importantly, our paper makes the important contribution that education levels appear to moderate the levels of CRP, opening the possibility for future studies.

Although these results yielded important conclusions, several limitations warrant consideration. The cross sectional nature of the data did not allow for establishing causality. A longitudinal design would support investigations that examine multiple factors implicated in transitions in intermediate and high CRP. Unfortunately, we were not able to test the heritability of plasma concentrations of CRP. Due to data limitations and restrictions on public use data, we were not able to include other variables that might confound the models such as genetics, neighborhood environments such as residential segregation and other health risk behaviors including diet. Despite these limitations, findings highlight multiple potential analytic associations between race/ethnicity, education and elevated CRP levels.

## 5. Conclusions

In conclusion, the present study extends research that seeks to understand the SES factors underlying race/ethnic disparities in inflammation. Our findings suggest the intersectional relationships among race/ethnicity and educational attainment are key associations that require scholarly attention to assure we address the measures that profoundly contribute to health disparities. As a significant body of work has identified the impact of stratification on our biomarkers, future studies should assess the role of neighborhoods as distal measures of SES that may play an integral part in elevating CRP and measure, prospectively, the role of oxidative stress to better understand how racial/ethnic stratification plays an important role in differentiating CRP risk. We provided empirical evidence of the importance of the education-inflammatory pathway for both NHBs and MAs that signals the need to focus on preventive detection of CVD risk for these populations that are over-represented in low SES environments, experience persistent chronic strains, and lack access to adequate health care.
