# Erratum: Dinwiddie, G.; *et al*. The Impact of Educational Attainment on Observed Race/Ethnic Disparities in Inflammatory Risk in the 2001–2008 National Health and Nutrition Examination Survey. *Int. J. Environ. Res. Public Health* 2016, *13*, 42

**DOI:** 10.3390/ijerph13040367

**Published:** 2016-03-29

**Authors:** International Journal of Environmental Research and Public Health Editorial Office

**Affiliations:** MDPI, Klybeckstrasse 64, Basel CH-4057, Switzerland; ijerph@mdpi.com; Tel.: +41-61-683-77-34

Due to an error during production, the legend of Figure 1 in the published paper [1] was incorrect. The correct figure is as follows:

We apologize for any inconvenience caused to readers or authors by these changes. The article will be updated and the original will remain available on the article webpage.

## Figures and Tables

**Figure 1:**
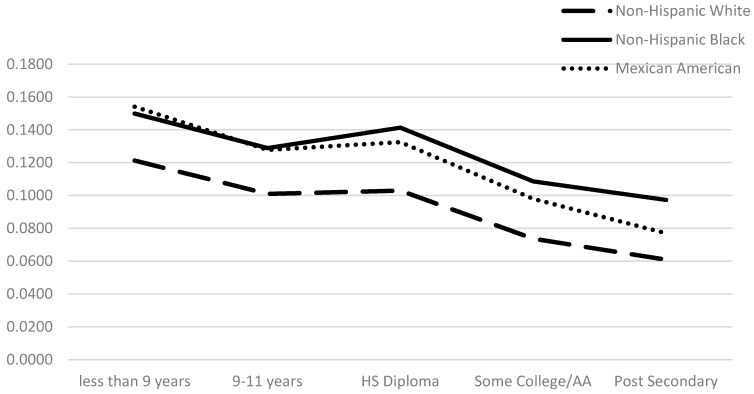
Predicted probability of intermediate c-reactive protein by education and race/ethnicity. Note: Differences across groups statistically significant at the *p* < 0.001 level. Model controls for age, diabetes, heavy drinker, HBP, HWC, income, sex, smoker, and statin use.
